# Astrocyte epigenetics as a priority area in neuroscience research

**DOI:** 10.3389/fnmol.2025.1716805

**Published:** 2026-01-12

**Authors:** Itzy E. Morales Pantoja, C. David Mintz

**Affiliations:** Department of Anesthesiology and Critical Care Medicine, The Johns Hopkins University School of Medicine, Baltimore, MD, United States

**Keywords:** astrocyte epigenetics, developmental disorders, epigenetic dysregulation, human astrocyte epigenetics, human *in-vitro* systems, neurodegeneration

## 1 Introduction

It took decades for the neuron-centric approach in neuroscience to appreciate the critical role that astrocytes play in health and disease. Now recognized as key regulators of brain function (Kwon et al., 2025; Santello et al., 2019), astrocytes' multifaceted roles mediate cellular crosstalk with neurons (Xie et al., 2022), oligodendrocytes (Zhang et al., 2025), and microglia (Faust et al., 2025), actively influencing human learning and behavior (Santello et al., 2019; Shigetomi and Koizumi, 2023). Underlying astrocytes' capacity to maintain balance and respond promptly is a flexible system of gene regulation, driven by a dynamic epigenetic landscape that adapts to immediate needs and shifts across a lifespan (Pavlou et al., 2019). Dysregulation of these malleable epigenetic programs may predispose the brain to pathological states. An emerging body of research suggests astrocytes play a central causative role in the pathology of neurodevelopmental and neurodegenerative disorders (Neal, 2018). In conditions such as autism spectrum disorder and Rett syndrome, alterations in astrocytic DNA methylation and histone modifications impact neuronal circuitry and plasticity (Marano et al., 2021). Similarly, in neurodegenerative diseases, shifts in astrocytic chromatin led to inflammatory and metabolic dysfunction (Neal, 2018; Celis et al., 2023).

We propose that astrocyte epigenetics offers a unifying framework in neuroscience; one that connects brain development, plasticity, and disease vulnerability, while highlighting upstream and potentially reversible mechanisms that could inform future therapeutic strategies.

## 2 Roles of astrocytes in brain development, cognition, and plasticity

Astrocytes are architects of brain development and function (Vivi and Di Benedetto, 2024; Williamson et al., 2024). They integrate developmental cues with adaptive responses, positioning them as central modulators of neural circuit formation and long-term brain function, contributing directly to the plasticity of learning, and regenerative capacity (Kozachkov et al., 2023; Hasel et al., 2025).

Astrocyte diversity may be best understood as a purposeful organizational feature of the CNS rather than a structural coincidence. The fact that protoplasmic and fibrous astrocytes occupy distinct gray and white matter domains, while specialized forms such as radial, Bergmann, Müller, velate, surface-associated, Gomori, and pituicytes arise in specific regions, suggests that astrocytes are tailored to the unique physiological demands of their local environments (Verkhratsky et al., 2023; Oberheim et al., 2012). The discovery of additional region-specific molecular subtypes through single-cell transcriptomic profiling strengthens this view, showing that astrocytes are not uniform support cells but exhibit spatially patterned gene expression programs that likely correspond to functional specialization (Kwon et al., 2025; Batiuk et al., 2020; Khakh and Deneen, 2019; Chai et al., 2017).

Given this deep functional integration, we propose that astrocyte epigenetics be prioritized as a central regulatory layer shaping developmental trajectories and cognitive outcomes.

## 3 Astrocytic epigenetic dysregulation in disease

### 3.1 Epigenetic immune memory

Astrocytic epigenetic immune memory is the ability of astrocytes to “remember” immune challenges, leading to chronic reactive states that amplify neuroinflammatory responses upon re-exposure (Lee et al., 2024). These states accelerate chronic neuroinflammation and disease progression in conditions such as AD, PD, ALS, and MS (Neal, 2018; Lee et al., 2024, 2023). This immune memory is encoded in epigenetic mechanisms that alter chromatin structure and gene expression. Multiple lines of evidence from single-cell genomics, functional perturbation experiments, and *in vivo* disease models converge to demonstrate that astrocytes acquire persistent reactive states encoding prior inflammatory exposures (Lee et al., 2024). Reactive astrocytes acquire a memory of inflammatory insults through ACLY- and p300-mediated DNA methylation changes. A consistent feature is cytokine-driven chromatin rewiring, particularly through IL-1α, TNF, and C1q, which reshapes astrocytic identity and function (Lee et al., 2024; Williamson and Deneen, 2024; Leng et al., 2022; Wischhof et al., 2024; Szebényi et al., 2021). These epigenetic changes profoundly alter astrocytic homeostasis, disrupting transitions into and out of reactive states, thereby contributing to disease progression (Lee et al., 2024; Cuautle et al., 2024; Ziff et al., 2022; McComish et al., 2024; Villarreal et al., 2021). For example, cell-specific CRISPR/Cas9 targeting ACLY+p300 in astrocytes in the EAE animal model of MS significantly reduced disease severity (Lee et al., 2024). CRISPRi (CRISPR interference) screens in human iPSC-derived astrocytes identified canonical NF-kB activation as a driver of inflammatory states, followed by IL-6 and interferon signaling (Diniz et al., 2025). Similarly, viewing reactivity as a spectrum rather than a binary A1/A2 switch better captures how astrocytes tune transcriptional programs to inflammatory cues (Linnerbauer et al., 2020; Sloan and Barres, 2014). This plasticity is rooted in development, as astrocytes transition from radial glia via gliogenic signals and intrinsic epigenetic programs, including DNMT1-mediated repression and later STAT3-p300/CBP-dependent activation of differentiation (Sloan and Barres, 2014). In mature astrocytes, cytokine-driven increases in MAFG homodimers, and MAT2A-dependent DNA methylation represses NRF2 signaling and stabilizes NF-κB-associated inflammatory phenotypes, supporting inflammatory memory (Linnerbauer et al., 2020). Thus, astrocyte identity is continuously shaped by developmental history and environmental experience, enabling functional diversity and persistent shifts toward reactive states when inflammation is sustained (Kwon et al., 2025; Khakh and Deneen, 2019).

Collectively, these findings show that persistent reactive states are not incidental, but encoded, reversible, and therapeutically actionable, arguing for systematic efforts to map, modulate, and reset epigenetic memory as a strategy to halt chronic neuroinflammation.

### 3.2 Neurodevelopmental dysfunction

Neurodevelopmental disorders are characterized by impaired brain maturation, disrupted neural connectivity, and cognitive and behavioral deficits. Although neurons have long dominated mechanistic discussions, astrocytes are key contributors to disease pathogenesis. Astrocyte development is orchestrated by dynamic chromatin remodeling, DNA methylation, and histone modifications that establish cell identity and regional specialization. Disrupting these epigenetic programs produces lasting alteration in astrocyte function (Lattke et al., 2021; Welle et al., 2021). Proper maturation requires the sequential activation of transcription factors that drive the transition from an immature to a mature state (Yu et al., 2025; Szewczyk et al., 2023). In developmental disorders, transcriptional dysregulation of these master regulators and their downstream networks shifts astrocytes toward reactive and inflammatory states, failing to provide appropriate metabolic, synaptic, and structural support. This leads to abnormal network formation and contributes to the cognitive and behavioral manifestations of disease (Szewczyk et al., 2023; Caldwell et al., 2022; Allen et al., 2022). Astrocytes reproducibly exhibit changes in DNA methylation, histone modifications, and chromatin remodeling in human tissue, iPSC-derived systems, and some animal models. These include MeCP2-dependent dysfunction, shifts in enhancer and histone acetylation, and the acquisition of epigenetic memory of reactivity. MeCP2, a chromatin organizer and transcriptional repressor that binds to methylated DNA, is notably linked to Rett syndrome, autism spectrum disorders, and X-linked intellectual disabilities (Tomasello et al., 2024; Bajikar et al., 2025). Human iPSC-derived astrocytes from individuals with ASD transplanted into the brain of healthy mice reduce neuronal network activity, spine density, and long-term potentiation (LTP), while inducing repetitive behaviors (Allen et al., 2022). Astrocyte alterations are a prominent feature of intellectual disability syndromes (Caldwell et al., 2022; Uguagliati and Grilli, 2024). In Down, Rett, and Fragile X syndromes, astrocytes exhibit altered secretome, including increased IGFBP2, which inhibits neurite outgrowth by suppressing IGF signaling in mouse models (Caldwell et al., 2022). Additionally, astrocyte transcriptional dysregulation of the BRD2-FG17 pathway is implicated in schizophrenia pathogenesis (Yu et al., 2025), while iPSC-derived astrocytes exhibited altered metabolic and inflammatory gene expression in schizophrenia (Szabo et al., 2021).

Taken together, these data position astrocytic epigenetic dysregulation as a causal driver of neurodevelopmental phenotypes. We propose that the field prioritize identifying developmental windows of astrocytic epigenetic vulnerability and leveraging patient-derived models to design targeted interventions.

### 3.3 Neurodegeneration and aging

Astrocyte chromatin remodeling and metabolic dysfunction are central mechanisms in neurodegeneration and aging. Two interconnected molecular systems govern astrocyte functional states: the epigenetic machinery (chromatin modifiers, DNA methylation enzymes, and histone-modifying complexes) and metabolic pathways (e.g., glycolysis, oxidative phosphorylation, amino acid, and lipid metabolisms) (Neal, 2018; Valori et al., 2019; Bonvento and Bolaños, 2021). In these bidirectional systems, metabolites serve as substrates and cofactors for chromatin remodeling, while chromatin states regulate the expression of metabolic genes (Mi et al., 2025). In neurodegeneration and aging, this chromatin-metabolism axis becomes dysregulated, creating pathological feedback loops that sustain inflammatory gene expression and suppress homeostatic programs (Diniz et al., 2025; Evans et al., 2022). Astrocytes in AD, ALS, and aging undergo coordinated chromatin remodeling and metabolic rewiring, keeping them in persistent inflammatory neurotoxic states (Cuautle et al., 2024; Villarreal et al., 2021; Diniz et al., 2025). This dysfunction is not merely a consequence of neuronal pathology but an active driver of disease progression (Evans et al., 2022; Jiwaji et al., 2022). Astrocyte vulnerability to pathological conversion is partially determined by early developmental programming (Lattke et al., 2021; Park et al., 2025), suggesting that neurodegenerative disease represents a late-life manifestation of early-established epigenetic vulnerabilities (Mi et al., 2025; Park et al., 2025). Multiple interventions, epigenetic modulators (Cuautle et al., 2024; Diniz et al., 2025), metabolic re-programmers (Spencer et al., 2025), activation blockers (Evans et al., 2022), and neuroprotective enhancers (Jiwaji et al., 2022), show preclinical efficacy, thus potentially ready for clinical translation. Success will require astrocyte-specific delivery technologies (Lattke et al., 2021), biomarkers for patient selection and monitoring (Valori et al., 2019), combination therapies targeting multiple nodes (Mi et al., 2025), and attention to disease stage and regional heterogeneity. Understanding and revising astrocyte-chromatin-metabolic dysfunction can shed light on the cycle of chronic neuroinflammation and neuronal loss (Evans et al., 2022; Jiwaji et al., 2022).

The chromatin-metabolism axis identifies astrocytes as mechanistic gatekeepers of neurodegenerative progression. We argue that future work should focus on developing astrocyte-specific epigenetic metabolic modulators to restore homeostatic programs in aging and disease.

## 4 Discussion: challenges and opportunities

### 4.1 Reproducibility of astrocyte epigenetic landscape in brain organoids

Brain organoids are a powerful tool for disease modeling and have opened the window into human-specific genetic developmental trajectories, including electrophysiological signatures (Lancaster and Knoblich, 2014; Qian et al., 2019; Trujillo et al., 2019). Limitations include that most systems best model early to mid-fetal stages of brain development, vary structurally, and lack robust vasculature and immune cell integration (Atamian et al., 2024; Bhaduri et al., 2020). Nonetheless, organoids remain the best bridge into the human brain. Abdolmaleky and colleagues (Marano et al., 2021; Mostafavi Abdolmaleky et al., 2024) demonstrated that iPSC-derived neurons and astrocytes from individuals with autism could recapitulate the molecular signatures of the disorder. Notably, these patient-derived cells mirrored postmortem findings in gene expression, epigenetic regulations, morphology, and connectivity. When applied to the study of astrocytic epigenetics, however, differences in the choice of epigenetic marks, disease models, and the depth of profiling directly affect reproducibility. The focus on the two canonical phenotypes A1 (neurotoxic) and A2 (neuroprotective) hinders the discovery of disease-specific, novel astrocytic subtypes.

Thus, improving reproducibility requires community standards for epigenetic profiling, controlled cell-state benchmarking, and the development of astrocyte-specific reference atlases across organoid platforms.

### 4.2 Disease modeling concordance

There is a growing consensus that many astrocytic epigenetic alterations are shared across neurodevelopmental disorders, e.g., Rett syndrome and autism spectrum disorders. These changes often converge on dysregulated inflammatory and synaptic pathways, with evidence pointing to aberrant chromatin remodeling, histone acetylation, and DNA methylation as common denominators. For instance, iPSC-derived astrocytes from individuals with autism, when transplanted into mice, were able to drive repetitive behaviors and cognitive decline through dysregulated calcium dynamics (Allen et al., 2022). Meanwhile, postmortem cortical transcriptomics showed upregulation of astrocytic immune programs and downregulation of synaptic genes (Gandal et al., 2022). Similar mechanisms are also found in Rett syndrome, where astrocytes show disrupted maturation and mitochondrial stress (Tomasello et al., 2024). Work in mouse knockouts, human stem cell-derived astrocytes, and *in vivo* models all point to astrocytes driving transcriptional, functional, and metabolic disturbances (Talvio and Castrén, 2024). However, many studies remain correlative, frequently relying on postmortem brain tissue. While this is an invaluable resource for gene expression analysis, postmortem studies are limited by confounding effects arising from global physiological responses to death. For example, activation of E2F transcription factors regulates both cell cycle progression and apoptosis (Ferreira et al., 2018; Iaquinta and Lees, 2007).

Thus, there is a need for integrated, longitudinal patient-derived studies that directly test causality and define cell-intrinsic astrocytic epigenetic mechanisms across neurodevelopmental and behavioral disorders.

### 4.3 Integration of multi-omics and single-cell technologies

The integration of multi-omics and single-cell technologies is reshaping the study of astrocytes by revealing their heterogeneity and the dynamic interplay between the epigenome, transcriptome, and other molecular layers. Single-cell multiomics enables the measurement of multiple modalities within the same cell, such as chromatin accessibility and gene expression, or scATAC-seq and scRNA-Seq, thereby linking epigenetic states directly to transcriptional activity (Lim et al., 2024; O'dea and Hasel, 2025; Burda et al., 2022; Miao et al., 2021). Spatial multiomics further improves this resolution by mapping astrocyte and molecular signatures within tissue context (Hennes et al., 2025; Sun et al., 2024). Overall, the field is shifting from broad population-level analysis to high-resolution, cell-specific characterization, providing detailed information with therapeutic potential for identifying disease-associated epigenomic signatures, and regulatory pathways to target reactive astrocyte subtypes. Understanding patient-specific astrocyte heterogeneity and variations in epigenetic regulation can support the development of personalized treatment strategies and patient stratification.

We propose the next phase of the field center to identify disease-relevant epigenomic regulators at single-cell resolution and to use these signatures to guide targeted therapeutic design.

### 4.4 A path toward translational astrocyte epigenetics

Rodent animal models have played a central role in demonstrating the biological relevance of astrocytic epigenetics, including chromatin regulation (Marano et al., 2021), enhancer remodeling (Serrano-Pozo et al., 2024), and inflammatory epigenetic memory (Lee et al., 2024; Linnerbauer et al., 2024). Yet it is human-derived *in vitro* systems that now offer the most direct window into patient-specific disease mechanisms. Recognizing this shift, in April 2025, the National Institutes of Health (NIH) and the Food and Drug Administration (FDA) advanced initiatives to reduce reliance on animal models by promoting New Approach Methodologies (NAMs). Building on this momentum, the NIH launched the Standardized Organoid Modeling (SOM) Center in September 2025 as a national resource to develop reproducible organoid platforms as alternatives for preclinical testing. Although current models are best suited to early development, the logical next step to expedite the translational potential will be to expand into region-specific brain organoids and larger constructs, such as assembloids, which could capture higher-order circuitry and astrocyte diversity. These platforms can provide a foundation for systematically assessing the astrocytic epigenetic landscape in a controlled and human-relevant context to understand how conserved molecular programs are modulated both regionally and disease-specifically. Though initially prioritizing reliability over complexity is necessary for achieving reproducibility, increasing complexity is essential for pursuing relevant and translational physiology. By integrating these models with multi-omic technologies and datasets to identify conserved regulators, it is possible to translate mechanistic insights into therapeutic strategies.

Ultimately, we argue that establishing rigorous, human-centric platforms for astrocyte epigenetics is essential for turning mechanistic insights into actionable therapeutic strategies and must become a priority across neuroscience research.
